# Association of Ki-67 Change Pattern After Core Needle Biopsy and Prognosis in HR+/HER2− Early Breast Cancer Patients

**DOI:** 10.3389/fsurg.2022.905575

**Published:** 2022-06-28

**Authors:** Shuai Li, Xiaosong Chen, Kunwei Shen

**Affiliations:** Department of General Surgery, Comprehensive Breast Health Center, Ruijin Hospital, Shanghai Jiaotong University School of Medicine, Shanghai China

**Keywords:** breast neoplasms, core needle biopsy, surgical sample, Ki-67 change, prognosis

## Abstract

**Background:**

To investigate the association of Ki-67 change pattern after core needle biopsy (CNB) and prognosis in HR+/HER2− early breast cancer patients.

**Method:**

Eligible patients were categorized into three groups: Low group, Elevation group, and High group. Chi-square test and logistic regression analysis were used to compare the clinic-pathological characteristics. Kaplan–Meier method was used to estimate the rates of recurrence-free interval (RFI) and breast cancer-specific survival (BCSS), which were compared *via* the Log-rank test. Cox proportional hazard analysis was performed to investigate independent prognostic factors.

**Results:**

A total of 2,858 patients were included: 1,179 (41.3%), 482 (16.9%), and 1,197 (41.8%) patients were classified into the low, elevation, and high groups, respectively. Age, tumor size, histological grade, lymph-vascular invasion (LVI), and ER level status were associated with Ki-67 change pattern after CNB. With a median follow-up of 53.6 months, the estimated 5-year RFI rates for the low group, elevation, and high groups were 96.4%, 95.3% and 90.9%, respectively (*P* < 0.001). And 5-year BCSS rates were 99.3%, 98.3% and 96.8%, respectively (*P* = 0.001). Compared with patients in the low group, patients in the high group had significantly worse RFI (hazard ratio [*HR*] 1.71, 95% confidence interval [*CI*] 1.16–2.54) in multivariate analysis.

**Conclusions:**

Ki-67 change after CNB was associated with prognosis in HR+/HER2− early breast cancer. Patients with Ki-67 high or elevation after CNB had an inferior disease outcome, indicating the necessity of re-evaluating Ki-67 on surgical specimens after CNB.

## Introduction

Core needle biopsy (CNB) has been widely accepted as a standard approach for the diagnosis and molecular subtyping of breast cancer (1–3). This facilitates informed patient discussion, one-step surgery, prognostic factors identification as well as (neo-)adjuvant therapy decision-making (1–3). However, several studies have reported discordance of estrogen receptor (ER), progesterone receptor (PR), human epidermal growth factor receptor 2 (HER2) and Ki-67 between preoperative CNB and surgical specimens (3–11).

Among these biomarkers, the Ki-67 index is a well-defined indicator for tumor proliferation and can be used as a prognostic factor for early breast cancer, particularly in those with HR+/HER2− diseases (12–14). Ki-67 is therefore used to distinguish luminal A-like versus luminal B-like breast cancer among HR+/HER2− diseases but not in the case of HER2+ or HR−/HER2− diseases (15). Ki-67 has also been included in multigene assays such as 21-gene recurrence score (Oncotype DX) which can predict prognosis and chemotherapy benefit among HR+/HER2− cancers (16–18). It, therefore, appears to be a promising marker for adjuvant chemotherapy use in this group of patients, which has been adopted by international guidelines (19–21). In the neoadjuvant setting, Ki-67 can be used as a surrogate of pCR to monitor treatment response and thus tailor decision-making in HR+/HER2− breast cancer, as in the IMPACT, POETIC, and WSG ADAPT HR+/HER2− trials (22–24). Moreover, Ki-67 ≥ 20% is used as an inclusion criterion in some clinical trials such as the MonarchE which evaluates the efficiency of an adjuvant CDK4/6 inhibitor in early high-risk HR+/HER2− breast cancer patients (25, 26).

However, Ki-67 was reported to display only a fair to moderate agreement between CNB and surgical specimens with a discordant rate of approximately 15% to 40% (5–11). It has been assumed that technical measurements, tumor heterogeneity, or tumor proliferation are driven by CNB might answer for the discordance (27–30). To date, however, there have been few studies reporting the association of Ki-67 change after CNB with disease outcomes in early HR+/HER2− breast cancer. Therefore, we performed this study to evaluate the accuracy of Ki-67 detection after CNB and the prognostic value of Ki-67 change pattern after CNB in this group of patients.

## Materials and Methods

### Patients

Patients who underwent CNB and surgery and were diagnosed with early invasive breast cancer at the Department of General Surgery, Comprehensive Breast Health Center, Ruijin Hospital, Shanghai Jiao Tong University School of Medicine from Jan 2009 to Mar 2020 were retrospectively analyzed.

Clinicopathological features, adjuvant systemic therapy and follow-up data were retrieved from Shanghai Jiao Tong University Breast Cancer Database (SJTU-BCDB). The main eligibility criteria were as follows: (1) invasive carcinoma; (2) no distant metastasis at diagnosis; (3) Ki-67 levels on CNB and surgical samples; (4) ER/PR-positive and HER2-negative. The exclusion criteria were: (1) male breast cancer; (2) prior cancer history; (3) neo-adjuvant therapy; (4) ER/PR- negative; (5) HER2-positive or unknown; (6) Ki-67 levels unknown.

### Pathology

In our institution, ultrasound-guided biopsies (4–6 per case) were taken with 14-gauge automated core biopsy needles. As described previously, pathological assessments of CNB and surgical samples were conducted by the Department of Pathology, Ruijin Hospital, Shanghai Jiao Tong University School of Medicine. Immunohistochemistry (IHC) and ﬂuorescence *in situ* hybridization (FISH), if necessary, of ER, PR, and HER2 were performed according to the ASCO/CAP guidelines (31–34). In brief, ER/PR positivity was defined as at least 1% tumor cells with nuclear staining in IHC and HER2 IHC 3+ or FISH+ was considered HER2+. To evaluate the Ki-67 index, cell distribution over the entire slice was first reviewed and 500–2000 cells were chosen from different microscopic views if the Ki-67 expression distribution was uniform. Otherwise, 2000 cells were equally counted in both hotspot and negative areas. Ki-67 expression was scored as the percentage of positive invasive tumor cells with any nuclear staining and recorded as the mean percentage of positive cells. The cut-off value to differentiate the low or high expression of Ki-67 was set as 20% (15, 35). Patients were categorized into the Low group if Ki-67 was low on both CNB and surgical samples, the Elevation group if Ki-67 was low on CNB but high on surgical samples, and the High group if Ki-67 was high on CNB.

### Follow-up

Patients were followed up at outpatient every 3 months in the first two years after surgery, every 6 months between 3rd and 5th years, and once every year thereafter until death. Recurrence-free interval (RFI) was calculated from the date of surgery to the date of local-regional recurrence, distant metastasis or death by breast cancer (36). Breast cancer-specific survival (BCSS) was calculated from the date of surgery to the date of death of breast cancer (36). The last follow-up time was April 5, 2021.

### Statistics

Clinicopathological characteristics and adjuvant therapy of the three groups were compared *via* Chi-square test or Fisher exact test. Factors with *P* < 0.05 were further evaluated in multivariate Logistic regression model. Rates of RFI and BCSS were estimated from Kaplan–Meier curves and compared *via* Log-rank test among the three groups. Cox proportional hazard regression models were used to investigate independent prognostic factors as well as to calculate hazard ratio (*HR*) and 95% confidence interval (95% *CI*). *P* < 0.05 was considered statistically significant. All statistical analyses were performed on SPSS (version 26.0).

## Results

### Baseline Clinicopathological Characteristics

**Figure 1:**
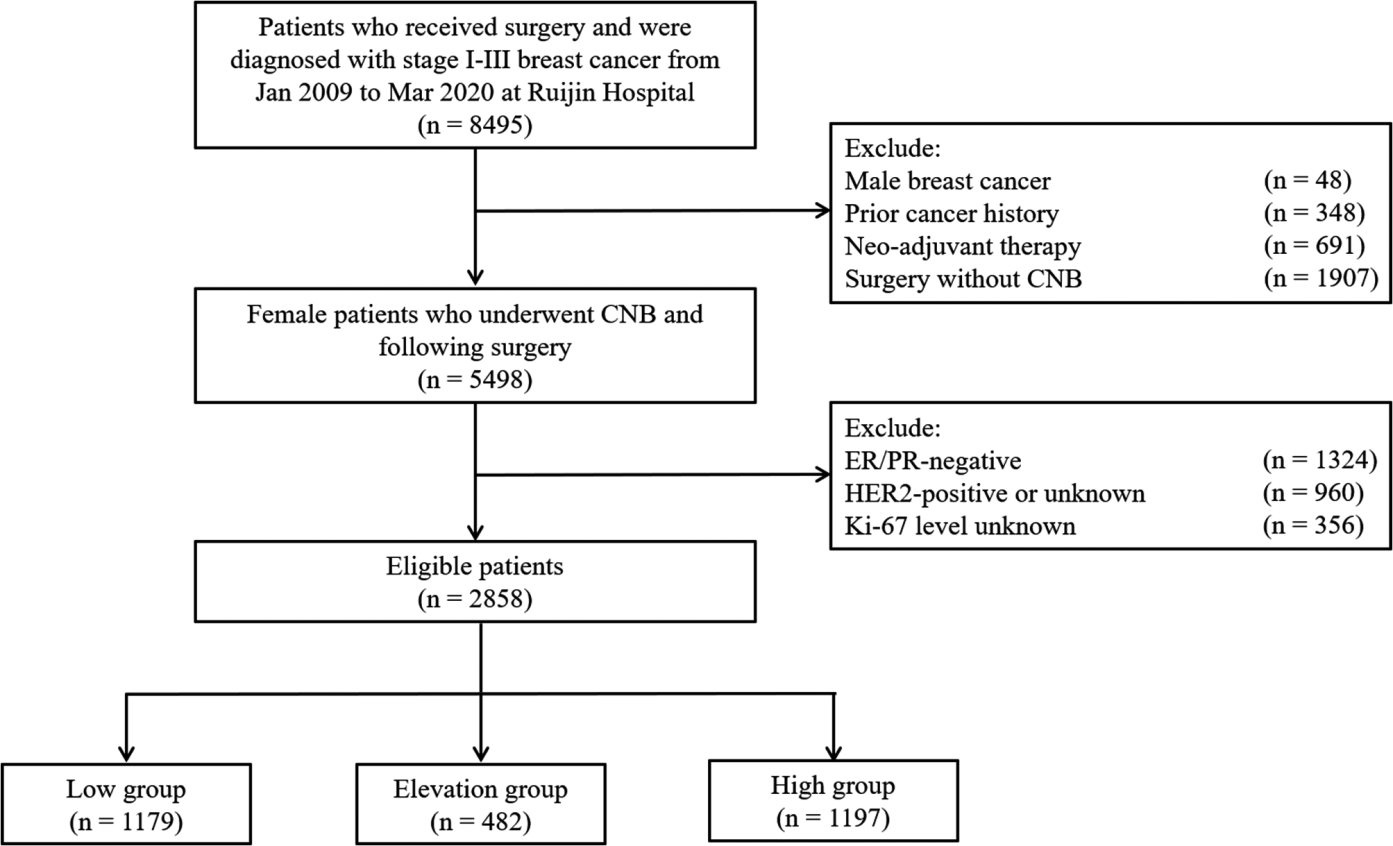
Flowchart of the study.

A total of 2,858 patients who underwent CNB and surgery and were diagnosed with HR+/HER2− early invasive breast cancer at the Department of General Surgery, Comprehensive Breast Health Center, Ruijin Hospital, Shanghai Jiao Tong University School of Medicine between January 2009 and March 2020 were included in the study (Figure 1). The baseline clinicopathological characteristics stratified by Ki-67 status are shown in Table 1. The median age was 57 (47–65) years old. There were 1,739 (60.8%) patients with tumors larger than 2.0 cm and 1,119 (30.2%) cases had positive ALNs. Ki-67 levels on CNB and surgical samples are summarized in Supplementary Figure S1. Among these patients, 1,179 (41.3%), 482 (16.9%), and 1,197 (41.8%) were classified as the Low group, the Elevation group, and the High group, respectively, with a concordance rate of 76.8% and a kappa value of 0.540. The median interval between CNB and surgery was 4 days, which were comparable between the groups (Supplementary Figure S2).

**Table 1:** Baseline clinical and pathological characteristics by Ki-67 change pattern after CNB.

Characteristics	Total, *N* = 2,858 (%)	Low, *N* = 1,179 (%)	Elevation, *N* = 482 (%)	High, *N* = 1,197 (%)	*P*-value
Age (y/o)					**<0.001**
<55	1,320 (46.2)	473 (40.1)	224 (46.5)	623 (52.0)	
≥55	1,538 (53.8)	706 (59.9)	258 (53.5)	574 (48.0)	
Breast surgery	** **	** **	** **	** **	**0.060**
BCS	961 (33.6)	419 (35.5)	169 (35.1)	373 (31.2)	
Mastectomy	1,897 (66.4)	760 (64.5)	313 (64.9)	824 (68.8)	
ALN surgery	** **	** **	** **	** **	**<0.001**
SLNB	1,722 (60.3)	794 (67.3)	286 (59.3)	642 (53.6)	
ALND	1,136 (39.7)	385 (32.7)	196 (40.7)	555 (46.4)	
Histology type	** **	** **	** **	** **	**<0.001**
IDC	2,506 (87.7)	986 (83.6)	427 (88.6)	1,093 (91.3)	
Non-IDC	352 (12.3)	193 (16.4)	55 (11.4)	104 (8.7)	
Tumor size	** **	** **	** **	** **	**<0.001**
<2.0 cm	1,119 (39.2)	555 (47.1)	182 (37.8)	382 (31.9)	
≥2.0 cm	1,739 (60.8)	624 (52.9)	300 (62.2)	815 (68.1)	
ALN status	** **	** **	** **	** **	**<0.001**
Negative	1,739 (60.8)	800 (67.9)	294 (61.0)	654 (53.9)	
Positive	1,119 (39.2)	379 (32.1)	188 (39.0)	552 (46.1)	
Histological grade	** **	** **	** **	** **	**<0.001**
I	224 (7.9)	189 (16.1)	16 (3.3)	19 (1.6)	
II	1,706 (59.9)	752 (64.1)	341 (70.9)	613 (51.3)	
III	602 (21.1)	65 (5.5)	70 (14.6)	467 (39.1)	
NA	318 (11.1)	168 (14.3)	54 (11.2)	96 (8.0)	
LVI	** **	** **	** **	** **	**<0.001**
Negative	2,515 (88.0)	1,108 (94.0)	426 (88.4)	981 (82.0)	
Positive	343 (12.0)	71 (6.0)	56 (11.6)	216 (18.0)	
ER level	** **	** **	** **	** **	**<0.001**
<50%	226 (7.9)	43 (3.6)	24 (5.0)	159 (13.3)	
≥50%	2,632 (92.1)	1,136 (96.4)	458 (95.0)	1,038 (86.7)	
PR	** **	** **	** **	** **	**<0.001**
Negative	328 (11.5)	95 (8.1)	42 (8.7)	191 (16.0)	
Positive	2,530 (88.5)	1,084 (91.9)	440 (91.3)	1,006 (84.0)	
Interval after CNB					**0.139**
<5 days	1,857 (65.0)	762 (64.6)	297 (61.6)	798 (66.7)	
≥5 days	1,001 (35.0)	417 (35.4)	185 (38.4)	399 (33.3)	
Chemotherapy	** **	** **	** **	** **	**<0.001**
No	1,064 (37.2)	677 (57.4)	155 (32.2)	232 (19.4)	
Yes	1,794 (62.8)	502 (42.6)	327 (67.8)	965 (80.6)	
Endocrine therapy	** **	** **	** **	** **	**0.002**
No	157 (5.5)	47 (4.0)	24 (5.0)	86 (7.2)	
Yes	2,701 (94.5)	1,132 (96.0)	458 (95.0)	1,111 (92.8)	

*Abbreviations: ALN, axillary lymph node; ALND, axillary lymph node dissection; BCS, breast-conserving surgery; CNB, core needle biopsy; ER, estrogen receptor; IDC, invasive ductal carcinoma; LVI, lymph-vascular invasion; NA, not available; PR, progesterone receptor; SLNB, sentinel lymph node biopsy; y/o, years old.*

### Baseline Characteristics According to Ki-67 Change Pattern After CNB

Age, histology type, tumor size, ALN status, histological grade, lymph-vascular invasion (LVI), ER level, and PR were significantly different among the low, elevation and high groups (*P* < 0.05, Table 1). In multivariate analysis, the overall distributions of age, tumor size, histological grade, LVI, ER level had significant differences among three groups (*P* < 0.05, Table 2). Compared with the Low group, patients in the High group were less likely to be age ≥55 years old (Odds ratio [*OR*] 0.64, *95% CI* 0.53–0.77), ER > 50% (*OR* 0.42, *95% CI* 0.28–0.65) and they had higher rates of tumors >2.0 cm (*OR* 1.38, *95% CI* 1.14–1.67), grade II disease (*OR* 6.94, *95% CI* 4.25–11.32), grade III disease (*OR* 50.48, *95% CI* 29.23–87.19), LVI (*OR* 2.38, *95% CI* 1.74–3.25) than patients in the Low group. Similarly, patients in the Elevation group were less likely to be age ≥55 years old (*OR* 0.76, *95% CI* 0.61–0.95) and more likely to have tumors >2.0 cm (*OR* 1.28, *95% CI* 1.02–1.61), grade II disease (*OR* 4.95, 95% *CI* 2.91–8.42), grade III disease (*OR* 11.08, 95% *CI* 5.97–20.61), LVI (*OR* 1.61, 95% *CI* 1.10–2.37).

**Table 2:** Patient baseline characteristics according to Ki-67 change pattern after CNB^a^.

Characteristics	Elevation		High		*P*-value
	*OR* (95% CI)	*P*	*OR* (95% CI)	*P*	
Age (y/o)	** **	** **	** **	** **	**<0.001**
<55	1.00		1.00		
≥55	0.76 (0.61–0.95)	0.017	0.64 (0.53–0.77)	<0.001	
Histology type	** **	** **	** **	** **	**0.088**
IDC	1.00		1.00		
Non-IDC	0.57 (0.29–1.10)	0.094	0.59 (0.34–1.02)	0.057	
Tumor size	** **	** **	** **	** **	**0.003**
<2.0 cm	1.00		1.00		
≥2.0 cm	1.28 (1.02–1.61)	0.037	1.38 (1.14–1.67)	0.001	
ALN status	** **	** **	** **	** **	**0.322**
Negative	1.00		1.00		
Positive	1.03 (0.81–1.31)	0.805	1.16 (0.95–1.41)	0.146	
Histological grade	** **	** **	** **	** **	**<0.001**
	1.00		1.00		
II	4.95 (2.91–8.42)	<0.001	6.94 (4.25–11.32)	<0.001	
III	11.08 (5.97–20.61)	<0.001	50.48 (29.23–87.19)	<0.001	
NA	6.00 (2.62–13.76)	<0.001	8.15 (3.97–16.72)	<0.001	
LVI	** **	** **	** **	** **	**<0.001**
Negative	1.00		1.00		
Positive	1.61 (1.10–2.37)	0.015	2.38 (1.74–3.25)	<0.001	
ER level	** **	** **	** **	** **	**<0.001**
<50%	1.00		1.00		
≥50%	0.78 (0.45–1.36)	0.383	0.42 (0.28–0.65)	<0.001	
PR	** **	** **	** **	** **	**0.183**
Negative	1.00		1.00		
Positive	1.05 (0.70–1.58)	0.805	0.78 (0.57–1.07)	0.126	

*Abbreviations: ALN, axillary lymph node; CNB, core needle biopsy; ER, estrogen receptor; IDC, invasive ductal carcinoma; LVI, lymph-vascular invasion; NA, not available; PR, progesterone receptor; y/o, years old.*

^a^ *The Low group was considered as the referee.*

### Ki-67 Change Pattern After CNB and Disease Outcomes

**Figure 2:**
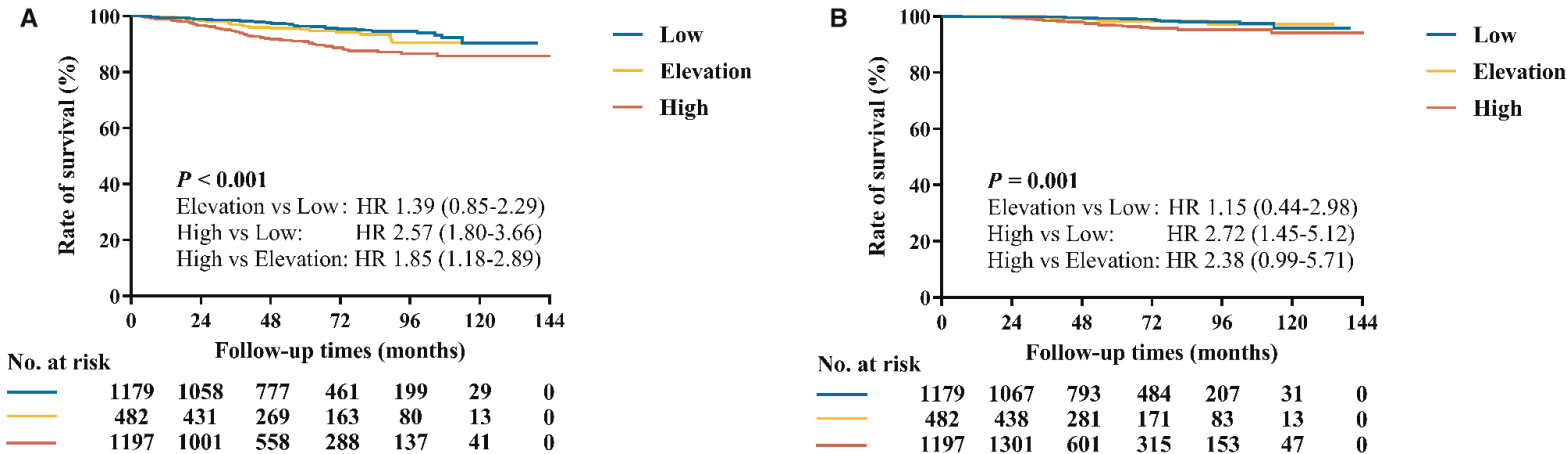
Kaplan–Meier curves of RFI and BCSS by Ki-67 change pattern after CNB. (**A**) The estimated 5-year RFI rates for the Low group, Elevation, and High groups were 96.4%, 95.3% and 90.9%, respectively (*P* < 0.001). (**B**) The estimated 5-year BCSS rates for the Low group, Elevation, and High groups were 99.3%, 98.3% and 96.8%, respectively (*P* = 0.001).

With a median follow-up of 53.6 (32.9–81.2) months, recurrences or deaths were observed in 187 patients (Supplementary Table S1). The estimated 5-year RFI rate was 96.4% for the Low group, 95.3% for the Elevation group, and 90.9% for the High group, respectively (*P* < 0.001, Figure 2A). Univariate analysis also identified that age, tumor size, ALN status, histological grade, LVI, ER level, PR were also associated with RFI (Supplementary Table S2). After adjusting these factors in multivariate analysis, the Ki-67 change pattern after CNB was still an independent prognostic factor for RFI (*P* = 0.022, Table 3). Compared with patients in the Low group, patients in the High group (hazard ratio [*HR*] 1.71, 95% *CI* 1.16–2.54) had significantly worse RFI.

**Table 3:** Multivariate Cox proportional regression analysis of prognostic factors affecting RFI, and BCSS.

Characteristics	RFI		BCSS	
	*OR* (95% CI)	*P*-value	*OR* (95% CI)	*P*-value
Age (y/o)		**0**.**007**		**/**
<55	1.00		/	
≥55	0.66 (0.48–0.89)		/	
Tumor size		**<0.001**		**0**.**080**
<2.0 cm	1.00		1.00	
≥2.0 cm	2.45 (1.61–3.73)		2.05 (0.95–4.42)	
ALN status		**<0.001**		**<0.001**
Negative	1.00		1.00	
Positive	2.03 (1.48–2.80)		4.03 (2.06–7.88)	
Histological grade		**0**.**080**		**0**.**974**
I	1.00		1.00	
II	1.40 (1.40–3.51)		0.82 (0.19–3.60)	
III	1.70 (0.65–4.46)		0.93 (0.19–4.50)	
NA	2.50 (0.93–6.73)		0.91 (0.15–5.63)	
LVI		**0**.**922**		**0**.**044**
Negative	1.00		1.00	
Positive	1.02 (0.64–1.63)		2.05 (0.95–4.42)	
ER level		**0**.**160**		**0**.**002**
<50%	1.00		1.00	
≥50%	0.70 (0.42–1.15)		0.32 (0.16–0.66)	
PR		**0**.**001**		**0**.**041**
Negative	1.00		1.00	
Positive	0.53 (0.36–0.76)		0.49 (0.25–0.97)	
Interval after CNB		/		**0**.**064**
<5 days	/		1.00	
≥5 days	/		1.68 (0.97–2.93)	
Chemotherapy	** **	**0**.**514**	** **	**0**.**183**
No	1.00		1.00	
Yes	1.17 (0.74–1.85)		1.96 (0.73–5.28)	
Endocrine therapy		**0**.**005**	** **	**0**.**287**
No	1.00		1.00	
Yes	0.51 (0.32–0.82)		0.62 (0.25–1.50)	
Ki-67 change pattern		**0**.**022**		**0**.**482**
Low	1.00		1.00	
Elevation	1.21 (0.73–2.00)		0.94 (0.36–2.47)	
High	1.71 (1.16–2.54)		1.41 (0.73–2.72)	

*Abbreviations: ALN, axillary lymph node; CNB, core needle biopsy; ER, estrogen receptor; IDC, invasive ductal carcinoma; LVI, lymph-vascular invasion; NA, not available; PR, progesterone receptor; y/o, years old.*

Regarding BCSS, 99.3% of patients in the Low group, 98.3% of patients in the Elevation group, and 96.8% of patients in the High group were still alive (*P* = 0.001, Figure 2B). Tumor size, ALN status, histological grade, LVI, ER level, and PR affected BCSS in univariate analysis (Supplementary Table S2). In multivariate analysis, however, the Ki-67 change pattern after CNB was not independently associated with OS (*P* = 0.482, Table 3).

## Discussion

Ki-67 index is a well-defined prognostic and predictive factor for early breast cancer, while few studies have focused on the prognostic value of Ki-67 change after CNB. Our study demonstrated more than 20% of patients would change the Ki-67 expression level after CNB. Moreover, the Ki-67 change pattern after CNB was associated with disease outcomes in HR+/HER2− early breast cancer. Patients with Ki-67 high or elevation after CNB had worse disease outcomes, indicating the necessity of re-evaluating Ki-67 on surgical specimens after CNB.

Ki-67 index is currently the most representative marker of tumor proliferation and thus an important indicator for therapeutic decisions in invasive breast cancer (12–14). CNB provides an important approach for the evaluation of Ki-67 level while results may vary using CNB and sequent surgical samples. Some explanations have been proposed for the discordant Ki-67 results obtained from CNB and surgical specimens (37–40). Tumor heterogeneity is one such explanation. A study of 353 patients confirmed that the diagnostic accuracy of CNB results increased as the number of harvested specimens increased (37). However, some other studies found that increasing CNB volume did not actually result in improved agreement rates of Ki-67 levels with surgical samples, indicating that there might be some other causes beyond heterogeneity (38, 39). Tumor proliferation driven by CNB might be one of these causes. For example, a significant increase of Ki-67 from biopsy to surgery was observed in HR-/HER2+ and triple-negative breast cancers by Focke and colleagues (39). Similarly, Chen et al. observed a 2.9% elevation of Ki-67 level after CNB and the change was associated with the surgery time interval between CNB and surgery as well as molecular subtype (40). This specific association suggests a real biological phenomenon such as an increase in cancer proliferation rather than an analytical artefact or a tissue sampling bias. However, the present study was unable to reveal the driving factors of Ki-67 change after CNB. Future studies may focus on topographical localizations of the Ki-67 expressing cells and expression levels of wound healing proteins to evaluate the association of wound healing reaction with Ki-67 change. Nonetheless, due to the positive results from the MonarchE study, Ki-67 ≥ 20% is necessary for choosing the adjuvant abemaciclib treatment in HR+/HER2− early breast cancer patients (25, 26). In our cohort, 16.9% of patients had Ki-67 level <20% in CNB but ≥20% on surgical specimens and they would potentially become candidates for adjuvant CDK 4/6 inhibitor.

In the present study, baseline characteristics including age, tumor size, histological grade, LVI, and ER level had significant differences among the three groups. Compared with patients whose Ki-67 remained low after CNB, patients in the Elevation group and the High group were younger and more likely to have grade II or III diseases and LVI. Moreover, patients in these groups tended to be ER <50% and tumor burdens were larger than those in the Low group. Taken together, the results suggested that high Ki-67 or Ki-67 elevation after CNB was associated with more aggressive clinical profiles, which was consistent with previous reports (5–11).

To date, few published articles have evaluated the prognostic value of Ki-67 change after CNB in untreated breast cancer patients (41). Previously, a small-sized study by Kristina A. Tendl-Schulz et al. showed that agreement of Ki-67 level between biopsy and surgery did not significantly impact recurrence-free survival (RFS) or OS (*P* = 0.22 for both) (41). Herein, we demonstrated in the large-scale cohort that Ki-67 change pattern after CNB was significantly associated with RFI and BCSS in HR+/HER2− breast cancers. Compared with the Low group, RFI for patients in the High group were significantly worse. More importantly, patients with Ki-67 elevation after CNB had numerically inferior survival compared to those patients whose Ki-67 remained at a low level. Though the prognostic roles of Ki-67 change were not satisfying enough in multivariate analysis and there might be other driving factors for the worse survivals beyond. The results did suggest an entity with more aggressive innate nature among those patients with low pre-surgical Ki-67 level and indicated that re-evaluation of Ki-67 level on surgical samples after CNB would provide more prognostic information.

Ki-67 change has been frequently used as a surrogate endpoint of treatment benefit and long-term outcome in the setting of “window of opportunity” presurgical trial (42–44). In the IMPACT trial, Ki-67 suppression following 2 and 12 weeks of endocrine treatment between the groups mirrored those in RFS in the ATAC (Arimidex, Tamoxifen Alone or in Combination) trial (22). Moreover, higher Ki-67 level after 2 weeks of endocrine therapy predicted significantly lower RFS (*P* = 0.004). In the POETIC Trial, postmenopausal women with ER+ breast cancer are randomized to a nonsteroidal aromatase inhibitor or to no treatment (2:1) for 2 weeks before surgery (23). The trial suggested that baseline Ki-67 (Ki-67_B_) and Ki-67 after 2 weeks (Ki-67_2w_) provided independent prognostic information. Both Ki-67_B_ and Ki-67_2w_ high expression was related with worse disease outcome. Patients with low Ki-67_B_ had good prognosis and only a minority of them had high Ki-67_2w_, suggesting no need for second Ki-67 measurement. In our cohort, the Ki-67 high group had the worst disease outcome, which was similar with the POETIC study. Moreover, our study found that 17% of patients had Ki-67 elevation after CNB with worse prognosis, which was different from the POETIC trial and the possible explanation were as follows: utilization of presurgical endocrine therapy, surgery time interval (4 days *versus* 2 weeks), case load (320 *versus* 32) as well as Ki-67 cutoff value (20% *versus* 10%).

We evaluated the prognostic value of the Ki-67 change pattern after CNB in a large-scale cohort of HR+/HER2− early breast cancer patients for the first time. However, there were several limitations in the present study. First, this was a single institutional retrospective study and there might be section bias in the results, which were therefore with limited generality. Further validation in other cohorts will provide us with more insights to the prognostic role of Ki-67 change after CNB. Moreover, the Ki-67 value was assessed after CNB in the surgical removal sample, which did not re-use the CNB during the radical surgical, which may cause the sampling bias. Thirdly, the present study was unable to answer whether wound healing reaction would have some effects on Ki-67 change after CNB. Last but not the least, the cutoff value for Ki-67 high or low expression level was not well defined, especially in the CNB samples.

In conclusion, our study demonstrated that Ki-67 change pattern after CNB was associated with disease outcomes in HR+/HER2− early breast cancer. Patients with high or elevating Ki-67 level after CNB had worse prognoses compared to those with both low Ki-67 value after CNB, indicating the necessity of re-evaluating Ki-67 level on surgical specimens after CNB.

## Data Availability

The data analyzed in this study is subject to the following licenses/restrictions: The database (SJTU-BCDB) can be applied by the participators only and all datasets included in the study accessible on reasonable requests. Requests to access these datasets should be directed to Xiaosong Chen, chenxiaosong0156@hotmail.com.
