# Supplementary material for: Association of Ki-67 Change Pattern After Core Needle Biopsy and Prognosis in HR+/HER2− Early Breast Cancer Patients
Source: Front Surg. 2022 Jun 28;9:905575. doi: 10.3389/fsurg.2022.905575 (PMC9275673; doi:10.3389/fsurg.2022.905575)
Supplement: Supplementary file 1 [file Table_1_v1.docx]

**Association of** **Ki-67 change pattern after core needle biopsy and prognosis in HR+/HER2- early breast cancer patients**

**Authors:**

Shuai Li ^1^, Xiaosong Chen ^1^*, Kunwei Shen ^1^*

**Affiliation:**

^1^ Department of General Surgery, Comprehensive Breast Health Center, Ruijin Hospital, Shanghai Jiaotong University School of Medicine, Shanghai 200025, China

**Correspondence to:**

Xiaosong Chen, 22nd Floor, 197 Ruijin Er Road, Shanghai 200025, China. E-mail: chenxiaosong0156@hotmail.com

Kunwei Shen, 22nd Floor, 197 Ruijin Er Road, Shanghai 200025, China. E-mail: [kwshen@medmail.com.cn](mailto:kwshen@medmail.com.cn)

**Supplementary Table S1** RFI and BCSS events by Ki-67 change pattern after CNB

|  | Total  N = 2858 (%) | Low  N = 1179 (%) | Elevation  N = 482 (%) | High  N = 1197 (%) |
| --- | --- | --- | --- | --- |
| **RFI events** |  |  |  |  |
| No recurrence or death of other reasons | 2671 (93.5) | 1123 (95.3) | 455 (94.4) | 1093 (91.3) |
| Local-regional recurrence | 52 (1.8) | 10 (0.8) | 7 (1.5) | 35 (2.9) |
| Distant metastasis | 111 (3.9) | 33 (2.8) | 17 (3.5) | 61 (5.1) |
| Death of breast cancer | 24 (0.8) | 13 (1.1) | 3 (0.6) | 8 (0.7) |
| **BCSS events** |  |  |  |  |
| Alive or death of other reasons | 2806 (98.2) | 1165 (98.8) | 476 (98.8) | 1165 (97.3) |
| Death of breast cancer | 52 (1.8) | 14 (1.2) | 6 (1.2) | 32 (2.7) |

Abbreviations: CNB, core needle biopsy; DFS, disease-free survival; OS, overall survival.

**Supplementary Table S2** Univariate analysis of prognostic factors affecting DFS, OS, and BCSS

| Characteristics | *P* value | |
| --- | --- | --- |
|  | RFI | BCSS |
| Age (< 55 y/o *vs.* ≥ 55 y/o) | 0.004 | 0.897 |
| Histology type (IDC *vs.* Non-IDC) | 0.891 | 0.266 |
| Tumor size (< 2.0 cm *vs.* ≥ 2.0 cm) | < 0.001 | 0.001 |
| ALN status (Negative *vs.* Positive) | < 0.001 | < 0.001 |
| Histological grade (Ⅰ *vs.* Ⅱ *vs.* Ⅲ *vs.* NA) | < 0.001 | 0.027 |
| LVI (Negative *vs.* Positive) | 0.012 | < 0.001 |
| ER level (< 50% *vs.* ≥ 50%) | < 0.001 | < 0.001 |
| PR (Negative *vs.* Positive) | < 0.001 | < 0.001 |
| Interval after CNB (< 5d *vs.* ≥ 5d) | 0.539 | 0.043 |
| Chemotherapy (No *vs.* Yes) | < 0.001 | < 0.001 |
| Endocrine therapy (No *vs.* Yes) | < 0.001 | 0.006 |
| Ki-67 change pattern  (Low *vs.* Elevation *vs.* High) | < 0.001 | 0.001 |

Abbreviations: ALN, axillary lymph node; CNB, core needle biopsy; ER, estrogen receptor; IDC, invasive ductal carcinoma; LVI, lymph-vascular invasion; NA, not available; PR, progesterone receptor; y/o, years old.

**

**

**Supplementary Figure S1** Ki-67 levels on CNB and surgical samples

**

**

**Supplementary Figure S2** Interval between CNB and surgery
